# Transcriptome characterization of three wild Chinese *Vitis* uncovers a large number of distinct disease related genes

**DOI:** 10.1186/s12864-015-1442-3

**Published:** 2015-03-21

**Authors:** Chen Jiao, Min Gao, Xiping Wang, Zhangjun Fei

**Affiliations:** State Key Laboratory of Crop Stress Biology in Arid Areas, College of Horticulture, Northwest A&F University, Yangling, Shaanxi 712100 China; Key Laboratory of Horticultural Plant Biology and Germplasm Innovation in Northwest China, Ministry of Agriculture, Northwest A&F University, Yangling, Shaanxi 712100 China; Boyce Thompson Institute for Plant Research, Cornell University, Ithaca NY, 14853 USA; USDA Robert W. Holley Center for Agriculture and Health, Tower Road, Ithaca NY, 14853 USA

**Keywords:** Grape, Chinese wild *Vitis*, *De novo* transcriptome, Disease related genes, *Cis*-NATs

## Abstract

**Background:**

Grape is one of the most valuable fruit crops and can serve for both fresh consumption and wine production. Grape cultivars have been selected and evolved to produce high-quality fruits during their domestication over thousands of years. However, current widely planted grape cultivars suffer extensive loss to many diseases while most wild species show resistance to various pathogens. Therefore, a comprehensive evaluation of wild grapes would contribute to the improvement of disease resistance in grape breeding programs.

**Results:**

We performed deep transcriptome sequencing of three Chinese wild grapes using the Illumina strand-specific RNA-Seq technology. High quality transcriptomes were assembled *de novo* and more than 93% transcripts were shared with the reference PN40024 genome. Over 1,600 distinct transcripts, which were absent or highly divergent from sequences in the reference PN40024 genome, were identified in each of the three wild grapes, among which more than 1,000 were potential protein-coding genes. Gene Ontology (GO) and pathway annotations of these distinct genes showed those involved in defense responses and plant secondary metabolisms were highly enriched. More than 87,000 single nucleotide polymorphisms (SNPs) and 2,000 small insertions or deletions (indels) were identified between each genotype and PN40024, and approximately 20% of the SNPs caused nonsynonymous mutations. Finally, we discovered 100 to 200 highly confident c*i*s-natural antisense transcript (*cis*-NAT) pairs in each genotype. These transcripts were significantly enriched with genes involved in secondary metabolisms and plant responses to abiotic stresses.

**Conclusion:**

The three *de novo* assembled transcriptomes provide a comprehensive sequence resource for molecular genetic research in grape. The newly discovered genes from wild *Vitis*, as well as SNPs and small indels we identified, may facilitate future studies on the molecular mechanisms related to valuable traits possessed by these wild *Vitis* and contribute to the grape breeding programs. Furthermore, we identified hundreds of *cis*-NAT pairs which showed their potential regulatory roles in secondary metabolism and abiotic stress responses.

**Electronic supplementary material:**

The online version of this article (doi:10.1186/s12864-015-1442-3) contains supplementary material, which is available to authorized users.

## Background

Grapes are among the most valuable fruit crops, grown on about 7 million ha with an annual production of approximately 67 million tonnes worldwide [1]. There are over 60 species of *Vitis* around the world [2] and *Vitis vinifera* is the most widely planted grapevine. However, most cultivars of *V. vinifera* are highly susceptible to various economically important diseases such as powdery mildew (PM) [3], downy mildew (DM) [4] and anthracnose [5]. Enhancing resistance to these diseases is the focus area in current grape breeding programs. Wild species may possess valuable genetic variations (e.g. new alleles, SNPs and indels) in disease resistance genes, particularly, in nucleotide-binding site leucine-rich repeat (NBS-LRR) proteins. It has been reported that the species-specific genes in wild and semi-wild watermelon were highly enriched with genes involved in disease-related processes [6]. In addition, the wild eggplant carries nearly 200 extra disease resistant genes compared to the cultivated eggplant [7]. Grape, unlike other domesticated crops, has retained high genetic diversity from wide progenitors [8]; however, most European cultivars are susceptible to many fungal diseases. Moreover, only limited disease resistant loci, such as *Run1* [9], *Ren1* [10], *Ren2* [11] and *Ren3* [12] that confer resistance to PM, and *Rpv* loci including *Rpv1* [13], *Rpv2* [14] and *Rpv3* [15] that confer resistance to DM, have been identified, mainly from wild grapevine species. Currently, the widely planted grape cultivars are very sensitive to diverse pathogens. Thus, genetic engineering of disease resistance in grape has become an increasing need and this can be facilitated by the use of wild *Vitis* resources.

China, as one of the major centers of the origin of *Vitis*, has more than 35 native *Vitis* species [16]. Chinese wild *Vitis* are naturally distributed throughout the country and many of them can survive in regions of high humidity and moisture [2], under which the occurrence of fungal diseases is increased [17]. A number of disease resistant Chinese wild *Vitis* have been identified and characterized. The pioneering work from Wang et al. [18] and Wan et al. [19] identified a large number of Chinese wild grapes that displayed strong resistance to PM. Wan et al. [19] also found about half of the Chinese wild *Vitis* were resistant to DM and around one third of them have both PM and DM resistances. In addition, Wang et al. [20] and Li et al. [21] found that all the investigated Chinese wild *Vitis* exhibited much less susceptibility to anthracnose compared to the two *V. vinifera* cultivars (Cabernet Sauvignon and Chardonnay). Moreover, Chinese wild *Vitis* showed other specific characteristics, such as high photosynthetic efficiency in *V. quiqangualaris* Rehd [22] and high content of resveratrol (a phytoalexin that is beneficial to human health) in *V. quiqangualaris* Danfeng-2 [23]. However, the underlying genetic variations contributing to these phenotypic differences have not yet been explored.

Grapevine is the first fruit crop that had its genome sequenced. The high quality genome has been served as the reference for many genetic studies. However, recent deep sequencing experiments have shown that relying on a single reference genome may underestimate the variability among different genotypes [24]. For example, reconstructing the transcriptomes of different grape genotypes has revealed substantial heterogeneity in transcripts that associated with their phenotypes [24,25]. In this study, we aim to characterize Chinese wild *Vitis* transcriptomes to explore the potential genetic diversities such as SNPs and indels. To this end, two Chinese wild *V. pseudoreticulata* accessions “Baihe-13-1” (BH) and “Hunan-1” (HN), and one *V. quinquangularis* accession “Shang-24” (S) were selected for deep transcriptome sequencing. The selected accessions possess valuable resistances to various fungal pathogens including PM (BH and S) [18], DM (BH and HN) [19] and anthracnose (BH, HN and S) [20]. We *de novo* assembled the transcriptomes and conducted comparative analysis of SNPs and small indels between wild accessions and the reference genome, PN40024. Distinct genes, which represent genes that are absent or highly divergent from sequences in the reference PN40024 genome, were then identified from each accession. Using transcript information from strand-specific RNA-Seq libraries, we have identified *cis*-natural antisense transcript (*cis*-NAT) pairs, which were known to participate in a broad range of regulatory events.

## Results

### Transcriptome sequencing, *de novo* assembly, and comparison with the reference genome

In order to more broadly capture disease related genes, we infected young leaves of the three Chinese wild grapes with PM. Based on the proposed PM infection cycle [26,27], as well as our previous studies [28-30], we collected leaves at 0, 6, 12, 24, 48, 72, 96 and 120 hours post inoculation (hpi). We then prepared eight independent strand-specific RNA-Seq libraries for each accession. In total, 24 libraries were constructed and sequenced on the Illumina HiSeq 2000 platform. After removing adaptors, low quality sequences, and ribosomal RNA (rRNA) reads (see method), we obtained a total of 53,890,427, 68,593,803 and 70,718,358 high quality cleaned reads for BH, HN and S, respectively (Table 1). The transcriptomes of the three wild Chinese *Vitis* were constructed *de novo* separately. The final assembled transcript sets of BH, HN and S contained 34,914, 38,528 and 38,204 contigs, respectively, with N50 lengths of 814 bp, 876 bp and 980 bp and average lengths of 602 bp, 630 bp and 679 bp (Table 1). The GC-contents of these three transcript sets were similar (~44.5%; Table 1), but slightly higher than that of the PN40024 transcripts (42.0%). The assembled transcriptome sequences of the three Chinese wild *Vitis* can be downloaded and blasted at http://bioinfo.bti.cornell.edu/wild_vitis.

**Table 1:** **Summary of transcriptome sequences and assemblies of the three Chinese wild** ***Vitis***

**Statistics**	**BH**	**HN**	**S**
**Sequences**			
Total raw reads	63,210,010	80,577,978	81,282,272
High-quality cleaned reads	53,890,427	68,593,803	70,718,358
High-quality cleaned nucleotides	2,911,785,932	3,703,865,895	3,864,462,091
**Assemblies**			
Number of assembled transcripts	34,914	38,528	38,204
Number of assembled bases	21,030,007	24,289,873	25,954,126
Contig N50	814	876	980
Average contig length	602	630	679
GC content (%)	44.7	44.5	44.4

BH, *V. pseudoreticulata* accession “Baihe-13-1”; HN, *V. pseudoreticulata* accession “Hunan-1”; S, *V. quinquangularis* accession “Shang-24”.

**Figure 1:**
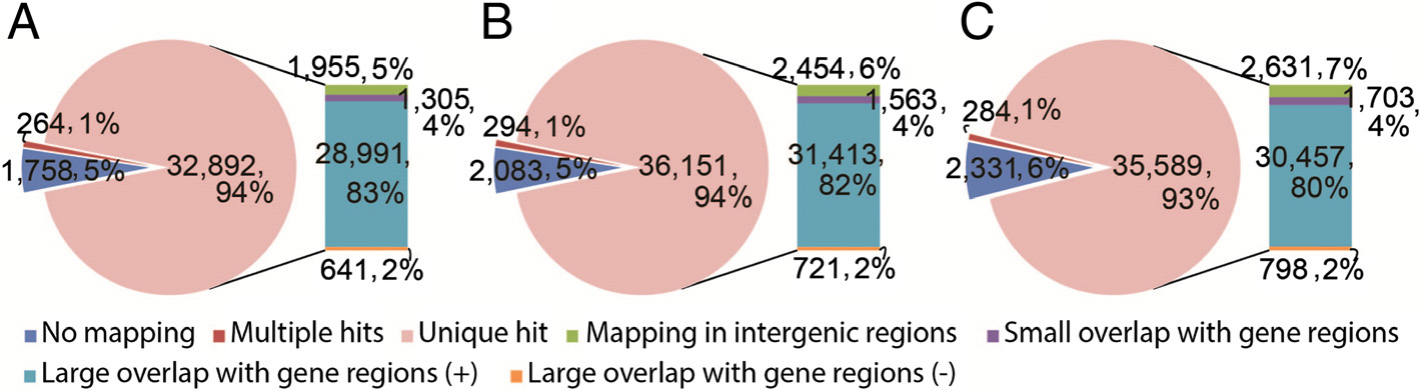
**Mapping of** ***de novo*** **assembled transcripts of the three Chinese wild** ***Vitis***, **BH (A), HN (B), and S (C), to the reference PN40024 genome.** No mapping: Contigs not mapped; Multiple hits: Contigs mapped to multiple genomic locations; Unique hit: Contigs mapped to unique genomic locations; Mapping in intergenic regions: Contigs mapped to intergenic regions; Small overlap with gene regions: Contigs mapped to gene regions with low overlapping (<90% of contig length); Large overlap with gene regions (+): Contigs mapped to gene regions in sense directions with high overlapping (≥90% of contig length) ; Large overlap with gene regions (−): Contigs mapped to gene regions in antisense directions with high overlapping (≥90% of contig length). BH, *V. pseudoreticulata* accession “Baihe-13-1”; HN, *V. pseudoreticulata* accession “Hunan-1”; S, *V. quinquangularis* accession “Shang-24”.

To discover the variations between Chinese wild *Vitis* and PN40024, a genotype derived from Pinot Noir, all three transcriptomes were aligned to the PN40024 genome. There were 94% (32,892), 94% (36,151) and 93% (35,589) of the total transcripts can be uniquely aligned to the reference genome with ≥ 97% sequence identity for BH, HN and S, respectively (Figure 1). Only 1% of them were mapped to multiple locations of the PN40024 genome and most of them were mapped to two locations. The majority of transcripts (83%, 82% and 80% of the total transcripts from BH, HN and S, respectively) were mapped to the annotated gene regions (version 12× V0) with overlap fraction ≥ 90% and had the same strand with the corresponding PN40024 transcripts; whereas around 2% of the transcripts were aligned to gene regions in antisense orientations. Notably, a small proportion (5-7%) of the transcripts were aligned entirely to the intergenic regions. Among all the assembled transcripts, 1,758 (5%, BH), 2,083 (5%, HN) and 2,331 (6%, S) could not be aligned to the PN40024 genome. We considered these transcripts as candidates for distinct genes.

### Functional annotation of wild Chinese *Vitis* transcripts

All the transcripts from the three Chinese wild *Vitis* were annotated by comparing their sequences against the TrEMBL and Swiss-Prot protein databases [31]. In all three genotypes, around 88.5% of the transcripts had hits in TrEMBL, and around 53.5% had hits in Swiss-Prot (Table 2). The high percentage of transcripts that hit to known proteins, combined with the high mapping rate to the reference genome, indicated the high quality of the *de novo* assembled transcripts.

**Table 2:** **Annotations of assembled transcriptomes of the three Chinese wild** ***Vitis***

**Database**	**BH**	**HN**	**S**
**TrEMBL**	31,022 (88.85%)	34,122 (88.56%)	33,809 (88.50%)
**Swiss-Prot**	18,704 (53.57%)	20,491 (53.18%)	20,703 (54.19%)
**GO term**	26,936 (77.15%)	29,491 (76.54%)	29,195 (76.42%)
**MetaCyc pathway**	3,114 (8.92%)	3,343 (8.68%)	3,208 (8.40%)

BH, *V. pseudoreticulata* accession “Baihe-13-1”; HN, *V. pseudoreticulata* accession “Hunan-1”; S, *V. quinquangularis* accession “Shang-24”.

The transcripts were further annotated by assigning them human-readable functional terms extracted from the functional descriptions of their homologous proteins using AHRD [32]. Approximately 80-81% of the transcripts from the three species could be assigned with functional terms. The Gene Ontology (GO) terms for each transcript were then extracted according to the annotations in Swiss-Prot and TrEMBL (Table 2). In total, about 77% transcripts from each accession could be assigned with GO terms from at least one of the three GO categories, biological process, cellular component and molecular function. The top five subcategories in biological process were “cellular process”, “biosynthetic process”, “response to stress”, “cellular component organization” and “nucleobase-containing compound metabolic process” (Additional file 1). Pathway analysis [33] revealed approximately 3,000 genes from each accession that involved in 505–513 biochemical pathways.

### Distinct genes identified in the three wild Chinese *Vitis*

**Figure 2:**
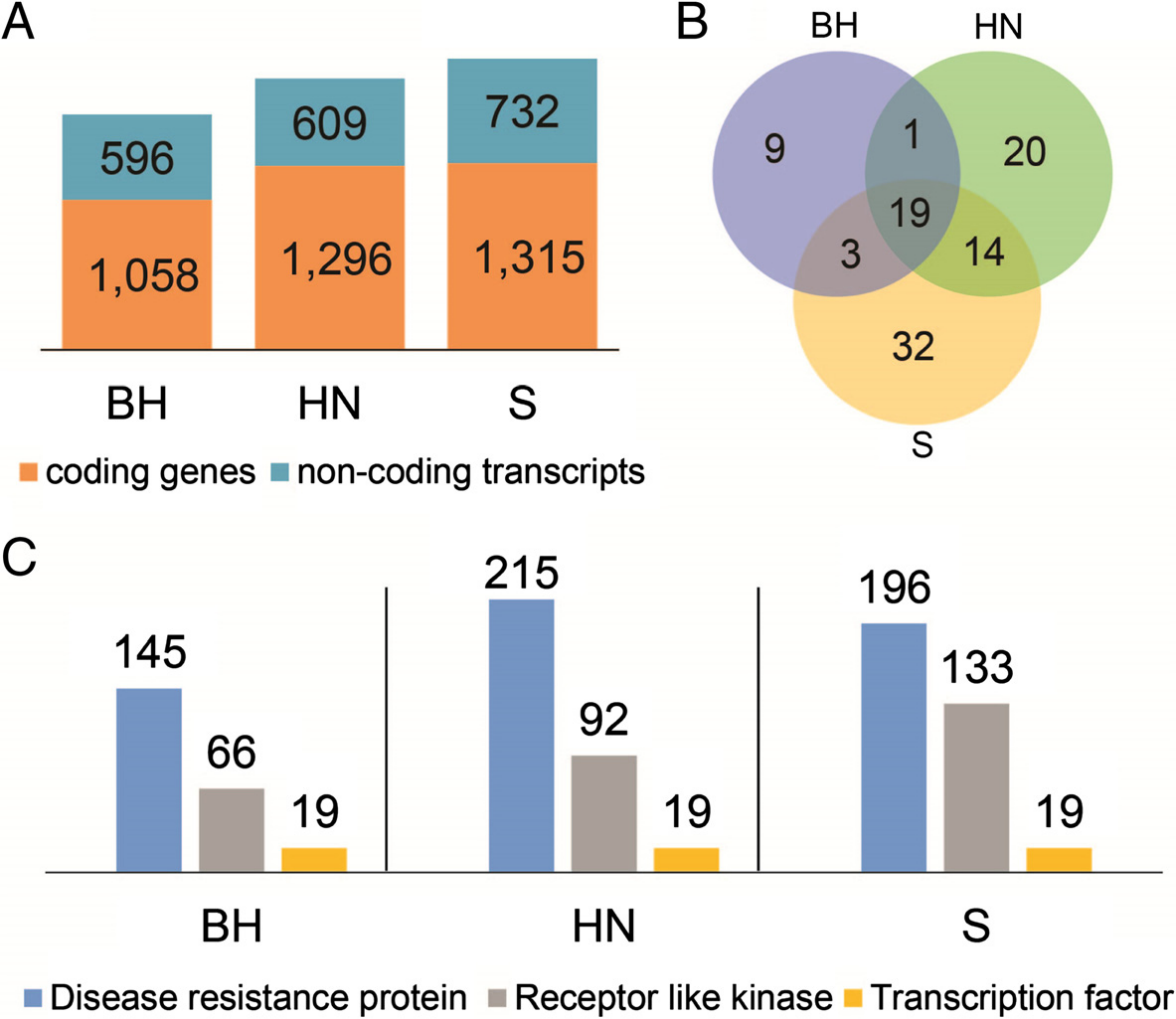
**Distinct genes in the three Chinese wild** ***Vitis*** **, BH, HN and S. (A)** Number of distinct protein-coding genes and non-coding transcripts from *de novo* transcriptome assemblies of the three Chinese *Vitis* leaf tissues, which were collected at 0, 6, 12, 24, 48, 72, 96, and 120 hours post inoculation with PM, respectively. **(B)** Venn diagram of GO terms enriched in the distinct protein-coding genes. **(C)** Number of distinct genes encoding disease resistance proteins, receptor like kinases and transcription factors. BH, *V. pseudoreticulata* accession “Baihe-13-1”; HN, *V. pseudoreticulata* accession “Hunan-1”; S, *V. quinquangularis* accession “Shang-24”.

The assembled transcripts included redundant sequences, mainly due to alternative splicing. We found that 4.80% (BH), 6.81% (HN) and 8.94% (S) transcripts could be clustered with other transcripts (sequence identity ≥ 97% and overlap length ≥ 100 bp). Only one representative transcript from each cluster was used in the downstream functional enrichment/annotation analysis, to avoid the repetitive counting of the same genes. Finally, we obtained a total of 1,650-2,000 distinct unique transcripts in each of the three accessions. The coding potential of these transcripts was then assessed by Coding Potential Calculator (CPC) [34]. Consequently, we got 1,058, 1,296 and 1,315 distinct genes with high coding potential from BH, HN and S, respectively, as well as 596, 609, and 732 potential non-coding transcripts (Figure 2A). The list of these transcripts is provided in Additional file 2. We then performed the GO term enrichment analyses (corrected P-value ≤ 0.05) on these three distinct protein-coding gene datasets. A total of 19 GO terms under the category of biological process were enriched in distinct genes in all three accessions (Table 3 and Figure 2B), and the most representative GO term was response to stimulus (GO:0050896), which included nearly half of the distinct genes. Among the child terms of GO:0050896, the most significantly enriched one was defense response (GO:0006952; Table 3). We also identified 15, 18 and 30 enriched child GO terms associated with resistance-related biological processes in BH, HN and S, respectively (Additional file 3).

**Table 3:** **GO terms enriched in the distinct gene sets of all three Chinese wild** ***Vitis***

**GO ID**	**GO term**	**GO term level**	**BH**			**HN**			**S**		
			C*	**B**	**Corrected P-value**	**C**	**B**	**Corrected P-value**	**C**	**B**	**Corrected P-value**
GO:0050896	response to stimulus	2	47%	35%	3.73E-13	50%	35%	8.09E-26	51%	35%	1.08E-27
GO:0006950	response to stress	3	38%	24%	1.65E-22	42%	24%	2.41E-45	41%	24%	9.38E-39
GO:0006952	defense response	4	26%	11%	4.26E-37	31%	12%	2.01E-78	31%	12%	2.34E-75
GO:0098542	defense response to other organism	4	11%	7%	2.01E-02	13%	7%	2.54E-09	14%	7%	3.21E-14
GO:0009617	response to bacterium	4	9%	5%	1.11E-03	10%	5%	5.25E-07	11%	6%	8.50E-11
GO:0045087	innate immune response	4	8%	4%	5.56E-04	8%	4%	4.34E-07	9%	4%	6.53E-11
GO:0006955	immune response	3	8%	4%	3.59E-03	8%	5%	7.25E-07	9%	5%	1.87E-10
GO:0042742	defense response to bacterium	6	8%	4%	5.15E-03	9%	5%	5.20E-09	10%	5%	4.03E-12
GO:0009814	defense response, incompatible interaction	5	4%	2%	9.50E-03	4%	2%	2.86E-04	4%	2%	7.07E-05
GO:0009816	defense response to bacterium, incompatible interaction	6	3%	1%	5.15E-04	3%	1%	3.91E-05	3%	1%	1.36E-05
GO:0009626	plant-type hypersensitive response	5	2%	1%	4.62E-02	3%	1%	8.40E-07	3%	1%	3.87E-06
GO:0098581	detection of external biotic stimulus	5	1%	0%	2.98E-02	1%	0%	1.03E-02	2%	0%	4.12E-11
GO:0009804	coumarin metabolic process	6	2%	0%	1.31E-03	2%	0%	1.84E-08	1%	0%	2.91E-03
GO:0009805	coumarin biosynthetic process	6	2%	0%	1.31E-03	2%	0%	1.84E-08	1%	0%	2.91E-03
GO:0015074	DNA integration	6	3%	0%	5.21E-23	2%	0%	5.65E-10	3%	0%	3.69E-15
GO:0015916	fatty-acyl-CoA transport	7	1%	0%	7.87E-08	0%	0%	3.88E-05	0%	0%	1.12E-03
GO:1901337	thioester transport	4	1%	0%	7.87E-08	0%	0%	3.88E-05	0%	0%	1.12E-03
GO:0080001	mucilage extrusion from seed coat	5	1%	0%	1.08E-05	1%	0%	2.57E-03	1%	0%	4.89E-03
GO:0009835	fruit ripening	5	1%	0%	4.40E-03	1%	0%	1.33E-05	1%	0%	1.52E-04

BH, *V. pseudoreticulata* accession “Baihe-13-1”; HN, *V. pseudoreticulata* accession “Hunan-1”; S, *V. quinquangularis* accession “Shang-24”.

*C: frequency of distinct genes belonging to the corresponding GO terms; B: frequency of all transcripts in each wild *Vitis* species belonging to the corresponding GO terms.

Plants produce three main secondary metabolites, terpenes, phenolics and nitrogen- and sulfur-containing compounds, which provide a major barrier against the attack of pathogens and herbivores [35,36]. Here, we found that several biological processes related to biosynthesis of phenolic compounds such as coumarin, were enriched in the distinct genes in all three accessions (Table 3). Biosynthesis of flavonoids, a class of phenolic compounds, were enriched in distinct genes of both BH and S. It is worth noting that callose deposition in cell wall and cell wall thickening biological processes and two other processes involved in the metabolism of anthocyanin-containing compounds were enriched in the distinct genes of the S accession. In addition, terpenoid compound metabolism and glucosinolate metabolic related processes were only enriched in the HN distinct genes (Additional file 3).

We found an important category of enriched biological processes that were related to the metabolism and action of plant hormones, a group of small molecules which function as versatile regulators of plant growth, development, reproduction and response to abiotic or biotic stresses [37,38]. Specifically, jasmonic acid related processes were enriched in distinct genes of BH and S, whereas ethylene biosynthetic and metabolic processes were overrepresented in distinct genes of both HN and S. Moreover, abscisic acid related process was only enriched in HN while processes related to salicylic acid and brassinosteroid were enriched in S distinct genes (Additional file 3).

We identified 145 (BH), 215 (HN) and 196 (S) distinct genes that were predicted to encode disease-related proteins (Figure 2C). Interestingly, a large number of them were NBS-LRR genes (81 in BH, 114 in HN and 107 in S), which play important roles in plant effector-triggered immunity (ETI) as they can directly or indirectly detect pathogen-associated proteins [39]. In addition, a large number of genes contained known protein domains related to disease resistance, such as NB-ARC [40], LRR, TIR and Dirigent [41]. Another group of distinct disease-related genes were those encoding receptor like protein kinases (66, 92 and 133 in BH, HN and S, respectively) (Figure 2C). In addition to the genes described above, we discovered several genes that are involved in plant-pathogen interaction, such as *Mlo*, *phytoalexin*-*deficient 4* (*PAD4*), *enhanced disease susceptibility 1* (*EDS1*), *RPW8.2* and genes encoding lipoxygenases [42-45].

Transcription factors play a central role in mediating biological activity in plant cells. We discovered 19 transcription factors in each of the three *Vitis* genotypes. More than 70% of these transcription factors belonged to the ethylene-responsive factor family, which has been reported to be involved in the control of primary and secondary metabolism, growth and developmental programs, as well as responses to environmental stresses [46].

### SNPs and small indels between wild Chinese *Vitis* and PN40024

**Figure 3:**
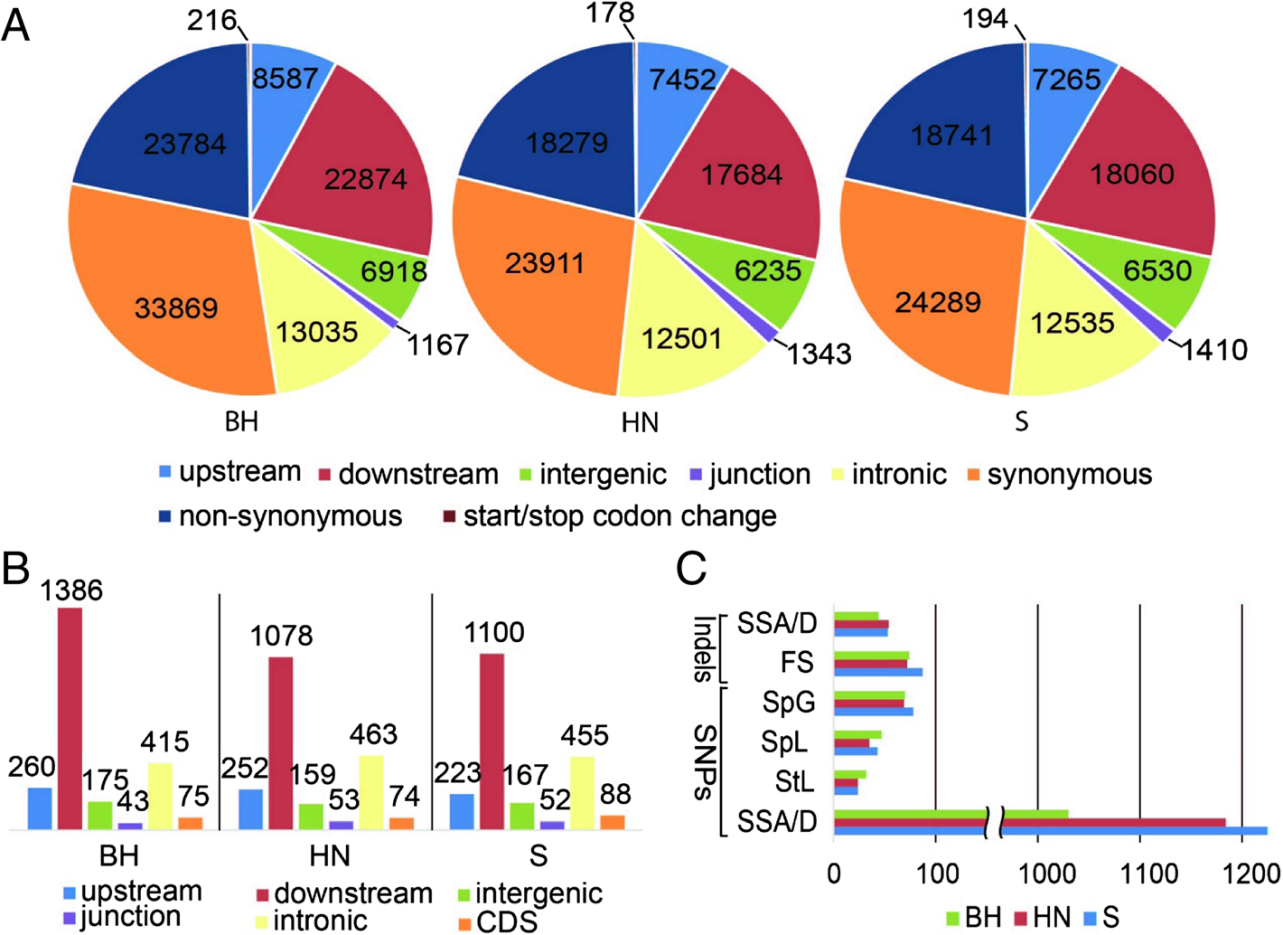
**SNPs and small indels between the three Chinese wild** ***Vitis*** **, BH, HN and S, and PN40024. (A)** Number of SNPs at different annotated regions of the reference PN40024 genome. **(B)** Number of small indels at different annotated regions of the reference PN40024 genome. **(C)** Number of genes affected by SNPs and small indels. SSA/D, splice site acceptor or splice site donor; FS, frame shift; SpG, stop codon gained; SpL, stop codon lost; StL, start codon lost. BH, *V. pseudoreticulata* accession “Baihe-13-1”; HN, *V. pseudoreticulata* accession “Hunan-1”; S, *V. quinquangularis* accession “Shang-24”.

Genomic variations, such as SNPs and small insertions and deletions (indels) are important driving force of genetic diversity. We identified SNPs and small indels through mapping the RNA-Seq reads from each accession to the reference PN40024 genome. We obtained a total of 110,450 SNPs and 2,354 small indels between BH and PN40024, 87,583 SNPs and 2,079 small indels between HN and PN40024, and 89,024 SNPs and 2,085 small indels between S and PN40024. Among these variations, 52% (BH), 48% (HN) and 49% (S) of SNPs, and 3.19% (BH), 3.56% (HN) and 4.22% (S) of small indels were located in the annotated coding regions (Figure 3A and 3B). In BH, we identified ~24,000 nonsynonymous substitutions, which potentially affect approximately 10,000 genes. In the other two accessions, we detected more than 18,000 nonsynonymous mutations that may alter the function of ~9,000 genes. The overall ratio of nonsynonymous to synonymous sites was ~0.7 in all three accessions. Interestingly, this ratio in NBS-LRR genes was substantially higher (1.3 in BH, 2.1 in HN and 3.0 in S). SNPs located at specific regions might have large effects on corresponding genes, such as gain or loss of start/stop codons, and disruption of splice site acceptors or donors [47,48]. We found 32, 24 and 24 genes in BH, HN and S, respectively, contained loss of start codon SNPs which would change the length of protein products (Figure 3C). About 40 genes with loss of stop codons and 70 genes with gain of stop codons were identified in each accession. Moreover, small indels mapped to coding regions (Figure 3B) led to the frame shifts of 74, 72 and 87 genes in BH, HN and S, respectively (Figure 3C). The identified SNPs and small indels were listed in Additional file 4.

### *Cis*-NATs in wild Chinese *Vitis*

Natural antisense transcripts (NATs) are endogenous RNA sequences partially or entirely complemented to other transcripts and *cis*-NAT pairs are two transcripts from the same genomic locus but on opposite strands [49]. We explored *cis*-NATs in our strand specific RNA-Seq data and identified 112, 145 and 196 *cis*-NAT pairs in BH, HN and S, respectively (Additional file 5). The classification of *cis*-NAT pairs based on their overlapping patterns was shown in Table 4. In addition, 84 (75.0%; BH), 115 (79.3%; HN) and 156 (79.6%; S) *cis*-NAT pairs were designated as coding-noncoding pairs depending on their coding potential calculated by CPC, while 25 (22.3%), 22 (15.2%) and 33 (16.8%) were classified as coding-coding pairs. *Cis*-NATs were previously reported to be involved in modulating various abiotic and biotic stresses [50,51]. Through functional analysis of the *cis*-NAT pairs we identified in the three Chinese wild grapes, we also found those related to abiotic stress responses were significantly enriched. In addition, *cis*-NATs involved in secondary metabolite biosynthetic processes were highly enriched; specifically, among the top five enriched biological processes, four (GO:0009698, GO:0009813, GO:0009812 and GO:0009699), three (GO:0009813, GO:0009812 and GO:0044550) and two (GO:0009813 and GO:0009812) enriched biological processes in BH, HN and S, respectively, were associated with flavonoid metabolic processes (Additional file 6).

**Table 4:** **Structure analysis of** ***cis*** **-NAT pairs in the three Chinese wild** ***Vitis***

**Category**	**Number of pairs**		
	**BH**	**HN**	**S**
Tail to tail (3’ to 3’)	31	43	58
Head to head (5’ to 5’)	28	47	63
Contained	53	55	75
Total	112	145	196

BH, *V. pseudoreticulata* accession “Baihe-13-1”; HN, *V. pseudoreticulata* accession “Hunan-1”; S, *V. quinquangularis* accession “Shang-24”.

## Discussion

In this study, we *de novo* assembled transcriptomes of three wild Chinese *Vitis* accessions. Distinct genes and genomic variations between Chinese *Vitis* and the reference PN40024 were extensively investigated. We identified a total of more than 1,000 distinct protein-coding genes, as well as 600–700 non-coding transcripts from each of the three Chinese wild *Vitis*. Almost half of the distinct protein-coding genes were functionally related to stimulus responses, and ~30% were involved in defense responses. The enrichment of stress-related processes in distinct genes is consistent with the hypotheses that wild plants, which are often exposed to an adverse environment, such as extreme temperatures, aberrant pH, toxic chemicals and pathogens, tend to have accelerated adaptive evolution of stress genes [52], while during domestication, a large number of stress and defense-related genes are lost in the cultivated species, which might be due to the many years of cultivation and selection that have focused on desirable fruit qualities at the expense of disease resistance [6].

Genomic variations, such as SNPs and indels, are important sources of genetic diversity. They are functionally significant and can cause phenotypic changes. Genetic analysis of plant disease resistance has shown that resistance is dominated by multiple loci and alleles at each locus are often highly polymorphic [53-55]. In the present study, we obtained 87,000-110,000 homozygous SNPs and ~2,000 small indels between each of the three wild Chinese *Vitis* and *V. vinifera*, genotype PN40024. This number is distinguished from a previous report on the identification of many fewer SNPs (~59,000) between *V. vinifera* cv. Corvina and PN40024 transcriptomes [24], probably due to both species belong to *V. vinifera*.

*Cis*-NATs are derived from the same genomic loci as their sense counterparts, but from the opposite strand. *Cis*-NAT pairs can be classified into three groups based on their overlapping patterns, including 3’ end overlap, 5’ end overlap and one entirely contained within the other [49]. In our study, across all three *Vitis* spp., the majority of *cis*-NAT pairs were those entirely contained within the other transcripts. This is consistent with the finding in soybean [56] but different from that in *Arabidopsis* [49], where most *cis*-NAT pairs have 3’ end overlap. *Cis*-NATs were reported to participate in plant responses to a wide range of abiotic stresses, such as cold [57] and salt [50] stresses. Consistent with these findings, we found that *cis*-NATs identified from wild Chinese grapes were significantly enriched with transcripts related to stress responses. In addition, we also observed high enrichment of secondary metabolism-related genes in the wild grape *cis*-NATs, implying the functional diversity of *cis*-NATs in mediating important biological processes in plants.

In the present study, we found 6-10% distinct genes were surface-localized receptor like kinases (RLKs). Some RLKs, as well as receptor like proteins (RLPs), are pattern-recognition receptors (PRRs) [58]. They contain various ligand-binding ectodomains that can perceive pathogen-associated molecular patterns (PAMPs), e.g. flagellin, peptidoglycans (PGNs) and chitins, or damage-associated molecular patterns (DAMPs). RLKs (e.g. LYK1) or RLPs (e.g. LYM2) can function independently or cohesively [59]. In this study, we discovered five distinct genes (four in S including one *LYK1* and one *LYM2* gene, and one in HN) containing the LysM motif, a common unit in both RLKs and RLPs that are responsible for binding to various types of PGNs and chitins. Unlike typical PRRs, Pep1 receptor (PEPR2) acts as a receptor for PEP defense peptides and senses an endogenous elicitor that potentiates PAMP-inducible plant responses [60]. One *PEPR2* was found in HN distinct genes. PEPR2 and its closest homolog PEPR1 can interact with BAK1 [61], which is a co-receptor for several PAMPs and regulates their function [58]. In addition, other RLKs that do not belong to PRRs, such as wall-associated kinase (WAK) also play important roles in plant immunity signaling pathway. In this study, we found 7–10 WAK family members in each distinct gene set. WAKs have an extracellular domain and they can interact with pectin and other proteins located in the cell wall. Induction of WAK1 is required for plants to survive during *P. syringae* infection. Furthermore, the increase of *WAK1* mRNA levels is part of the defense response caused by exposure to jasmonic acid (JA), ethylene, or fungi [62]. A previous study has unraveled adaptive evolution of extracellular domains of RLKs [63], which may explain why many RLKs and RLPs were observed in distinct genes. The discovery of these genes may also imply the functional conservation in response to pathogen invasion.

Pathogens can overcome PAMP-triggered immunity (PTI) by deploying effectors to interfere with the PTI activated signaling pathways. These effectors can be perceived by plant R genes (mainly NBS-LRR) and activate plant immune system, also known as effector-triggered immunity. These pathogens are usually highly specialized for specific host plants, and the interaction at the molecular level is often complicated because of the co-evolution of the host and pathogens [64]. Wild and domesticated plant species have been exposed to the natural selection forces, and show divergent R genes as they have to initiate an arms race with the pathogen effector. However, compared to clonally propagated grape cultivars, wild *Vitis* may have evolved new disease resistance genes during sexual propagation. Several studies have demonstrated that in plant R-gene products, LRR domains are the major determinants of recognition specificity for effectors [65] and these domains were under diversifying selection to increase amino acid variability. Mechanisms for the evolution of new specificities are flexible, such as gene conversion and unequal recombination, as well as accumulation of amino acid codon exchanges in members of anciently duplicated gene families [66]. In our distinct genes, ~8% were NBS-LRR genes. Notably, from our SNP analysis, we found an R gene, GSVIVT01032161001, which has longer protein product in the three wild *Vitis* compared with that in PN40024 caused by a SNP in its stop codon. The longer R gene might confer increased specificity for pathogen recognition in the three Chinese wild *Vitis* species. Interestingly, in a wild potato, a homolog of the *Vitis* longer R gene has been identified, which is heterozygous (six amino acids loss in the 18^th^ LRR repeat for one allele) and confers broad spectrum resistance to late blight [67].

In this study, a number of flavonoid and isoprenoid biosynthesis genes were identified in the distinct gene sets. Flavonoid and isoprenoid biosynthesis was found to be up-regulated in both *V. vinifera* and *V. pseudoreticulata* that were inoculated with powdery mildew [27,68]. We also found *cis*-NATs were enriched with flavonoid biosynthesis-related genes in all three wild grapes, which may suggest that flavonoid metabolism is regulated by *cis*-NATs. In our distinct gene sets, several genes related to coumarin biosynthesis and metabolism were discovered. Coumarin, a known phytoalexin, was found to accumulate in parsley cells treated with a fungal glucan elicitor [69]. Several other phytoalexin-related genes were also found in our distinct gene sets, including stilbene synthase (STS) which is a key gene in the biosynthesis of stilbenes, and *phytoalexin*-*deficient 4* (*PAD4*), a well-known resistance gene in Arabidopsis. A recent study showed that Arabidopsis transformed with *Vitis enhanced disease susceptibility 1* (*EDS1*) and *PAD4* did not display rescued resistance to powdery mildew, even though these two proteins interacted when transiently expressed in *Nicotiana benthamiana*. Therefore, involvement of additional interacting proteins might be necessary for resistance to occur [70].

Transcriptional regulation of stress responsive genes plays a central role in abiotic/biotic stress responses. ERF, a subfamily of AP2 transcription factor, has been reported to be involved in many stress responses [71]. Pti4, an ERF in tomato, can be phosphorylated by a disease resistance protein (Pto) and regulate GCC-box PR genes [72]. McGrath et al. [73] found that AP2/ERF genes were the predominant transcription factor family genes responsive to both JA and a fungal pathogen *Alternaria brassicicola* in Arabidopsis. Specifically, AtERF2 acted as a positive regulator of JA-responsive defense gene expression and resistance to *A. brassicico* while AtERF4 showed the opposite functions. Their results suggest that plants coordinately express multiple repressor- and activator-type AP2/ERFs during pathogen challenge to modulate defense gene expression and disease resistance [73]. Interestingly, in our distinct gene sets from each of three wild species, 70% of the transcription factors belonged to the AP2/ERF family, which suggest that in Chinese wild *Vitis* the distinct AP2/ERF genes might co-evolve with R genes and have a high degree of sequence variations.

## Conclusions

In the present study we *de novo* constructed transcriptomes of three Chinese wild grapes, which showed resistances to various fungal pathogens. A comprehensive comparison between these transcriptomes and the reference grape genome unraveled a large number of distinct genes and a rich resource of genetic variations such as SNPs and small indels. Interestingly, many genetic divergences between wild and cultivated grapes were found to be highly related to many important biological processes, particularly defense associated processes, suggesting that the accelerated evolution of these genes may contribute to plant adaptation to different environments. Furthermore, the significant enrichment of *cis*-NAT pairs related to secondary metabolism and abiotic stress responses may shed lights on the potential regulatory roles of *cis*-NATs in Chinese wild *Vitis*.

## Methods

### Plant material, PM inoculation and RNA-Seq library preparation

Two Chinese wild *V. pseudoreticulata* accessions “Baihe-13-1” and “Hunan-1”, and one *V. quinquangularis* accession “Shang-24”, were maintained in the grape germplasm resource orchard at Northwest A&F University, Yangling, China (34° 20’ N, 108°24’ E). Young leaves from three separate vines of each accession were inoculated with PM [*Erysiphe necator* (Schw.) Burr.] as previously described [18]. *E. necator* as an obligate biotrophic fungus, grows and reproduces only on living grapes. The isolate we used was obtained from grape leaves showing fully developed PM symptom. To collect samples that had a good represntation for the PM infection process, which would more broadly capture disease related genes responsive to PM infection, we harvested leaves at 0, 6, 12, 24, 48, 72, 96 and 120 hpi based on the proposed PM infection cycle [26,27] and our previous studies [28-30]. The collected leaves were immediately frozen in liquid nitrogen and stored at −80°C till use. Total RNA was extracted following the method described in Guo et al. [30]. The quality and quantity of RNA were assessed by electrophoresis on 1% agarose gels and by a NanoDrop 1000 spectrophotometer (Thermo Scientific, Wilmington, DE, USA), respectively. Strand-specific RNA-Seq libraries were constructed using the protocol described in Zhong et al. [74] and sequenced on the Illumina HiSeq 2000 platform using the single-end mode.

### Data processing, *de novo* assembly and comparison with the reference genome

Raw reads from each of the three Chinese wild *Vitis* accessions were processed using Trimmomatic [75] to remove adaptor and low quality sequences. Reads shorter than 40 bp were discarded. The resulting reads were aligned to the ribosomal RNA database [76] using bowtie [77] and those aligned were discarded. The resulting high-quality cleaned reads (final reads) were subjected to Trinity [78] for *de novo* assembly with the minimum kmer coverage set to two. The final reads were then aligned to the assembled contigs using bowtie [77]. To remove false transcripts with antisense direction which was due to the incomplete digestion of the 2^nd^ strand during the strand-specific RNA-Seq library construction [74], contigs with the number of reads aligned in sense direction less than 1/10 of the number of reads aligned in antisense direction were discarded. The assembled contigs were then compared against the GenBank nt database [79], and those having hits from viruses, bacteria, archaea and fungi, but not from plant species, were removed. The final set of assembled contigs were further clustered to remove redundancies using iAssembler [80] with sequence identity cutoff set to 97%. Finally, we used seqclean [81] to trim polyA tails and to remove rRNA sequences at the contig level. The final assembled transcripts were aligned to the 12 × PN40024 genome assembly [82] using BLAT [83] with sequence identity no less than 97%.

### Functional annotation of assembled transcripts

To annotate the assembled transcripts, their sequences were searched against the TrEMBL and Swiss-Prot [31] protein databases using the blastx program, with the E-value cutoff of 1e-10. GO terms were assigned to the assembled transcripts based on the GO terms assigned to their hits in TrEMBL and Swiss-Prot databases [84]. The functional terms were assigned to each transcript using automated assignment of human readable descriptions (AHRD) [32]. Enzyme-encoding genes were extracted based on the AHRD result, and used to predict biochemical pathways using the Pathway Tools software [33].

### Identification of distinct genes

We first clustered the assembled transcripts under the criteria, sequence identity ≥ 97% and overlap length ≥ 100 bp, to remove redundant transcripts that could be derived from the same gene locus, mainly due to alternative splicing. The longest representative transcript from each cluster was kept and then aligned to the PN40024 genome [82] using BLAT [83] with sequence identity ≥ 97%. The unmapped transcripts, which were considered as distinct transcripts, were then checked for their coding potential using Coding Potential Calculator (CPC) [34]. Transcripts with positive coding potential scores were identified as distinct protein-coding genes and those with negative scores were non-coding transcripts. GO term enrichment analysis of the distinct protein-coding genes was performed using GO::TermFinder [85]. The putative functional domains from Pfam database [86] were identified for these distinct genes using HMMER3.0 [87].

### Identification of SNPs and small indels

To identify SNPs and small indels between each of the three Chinese wild accessions and the grape reference, raw RNA-Seq reads were aligned to the grape reference genome using the Burrows-Wheeler Aligner [88]. Only one of the duplicated RNA-Seq reads was kept to minimize the artifacts of PCR amplification, and only reads uniquely mapped to the genome were kept. Following mapping, SNPs and small indels were identified based on the mpileup files generated by SAMtools [89]. The identified SNPs and small indels were supported by at least four distinct RNA-Seq reads and had an allele frequency > 70%. The effects of detected SNPs and small indels were analyzed based on the 12 × V0 annotation of the PN40024 genome.

### Identification of *cis*-NATs

To identify *cis*-NAT pairs, we first compared the sequences of assembled transcripts from each wild *Vitis* accession against themselves. Transcript pairs which were aligned in reverse direction and had the overlap length greater than 50 bp were kept. The resulting transcript pairs were then aligned to the reference grape genome and those aligned to the same genome regions and showing distinct splicing patterns were identified as *cis*-NAT pairs.

### Availability of supporting data

The raw sequencing data has been deposited in NBCI SRA under the accession numbers SRP051051, SRP051054 and SRP051078: http://www.ncbi.nlm.nih.gov/sra/?term=SRP051051, http://www.ncbi.nlm.nih.gov/sra/?term=SRP051054, http://www.ncbi.nlm.nih.gov/sra/?term=SRP051078.

## Additional files

Additional file 1:
**GO classification of assembled transcriptomes of the three Chinese wild** ***Vitis*** **.** The figure shows GO term classification of assembled transcripts of the three Chinese wild *Vitis*, *V. pseudoreticulata* accession “Baihe-13-1” (BH), *V. pseudoreticulata* accession “Hunan-1” (HN) and *V. quinquangularis* accession “Shang-24” (S). Additional file 2:
**Distinct genes identified in the three Chinese wild** ***Vitis*** **.** The table provides the lists of distinct genes identified in the three Chinese wild *Vitis*, *V. pseudoreticulata* accession “Baihe-13-1” (BH), *V. pseudoreticulata* accession “Hunan-1” (HN) and *V. quinquangularis* accession “Shang-24” (S). Additional file 3:
**GO terms enriched in the distinct genes from the three Chinese wild** ***Vitis*** **.** The table provides the lists of GO terms that were enriched in the distinct genes from the three Chinese wild *Vitis*, *V. pseudoreticulata* accession “Baihe-13-1” (BH), *V. pseudoreticulata* accession “Hunan-1” (HN) and *V. quinquangularis* accession “Shang-24” (S). Additional file 4:
**SNPs and small indels between each of the three Chinese wild** ***Vitis*** **.** The table provides the lists of SNPs and small indels between the three Chinese wild *Vitis*, *V. pseudoreticulata* accession “Baihe-13-1” (BH), *V. pseudoreticulata* accession “Hunan-1” (HN), *V. quinquangularis* accession “Shang-24” (S), and *V. vinifera* PN40024. Additional file 5:
***cis*** **-NAT pairs identified in the three Chinese wild** ***Vitis*** **.** The table provides the lists of *cis*-NAT pairs identified in the three Chinese wild *Vitis*, *V. pseudoreticulata* accession “Baihe-13-1” (BH), *V. pseudoreticulata* accession “Hunan-1” (HN) and *V. quinquangularis* accession “Shang-24” (S). Additional file 6:
**GO terms enriched in** ***cis*** **-NATs from the three Chinese wild** ***Vitis*** **.** The table provides the lists of GO terms that were enriched in *cis*-NATs from the three Chinese wild *Vitis*, *V. pseudoreticulata* accession “Baihe-13-1” (BH), *V. pseudoreticulata* accession “Hunan-1” (HN) and *V. quinquangularis* accession “Shang-24” (S).
