# Supplementary material for: Transcriptome characterization of three wild Chinese Vitis uncovers a large number of distinct disease related genes
Source: BMC Genomics. 2015 Mar 21;16(1):223. doi: 10.1186/s12864-015-1442-3 (PMC4373064; doi:10.1186/s12864-015-1442-3)
Supplement: Additional file 1: — GO classification of assembled transcriptomes of the three Chinese wild Vitis . The figure shows GO term classification of assembled transcripts of the three Chinese wild Vitis, V. pseudoreticulata accession “Baihe-13-1” (BH), V. pseudoreticulata accession “Hunan-1” (HN) and V. quinquangularis accession “Shang-24” (S). [file 12864_2015_1442_MOESM1_ESM.pdf]

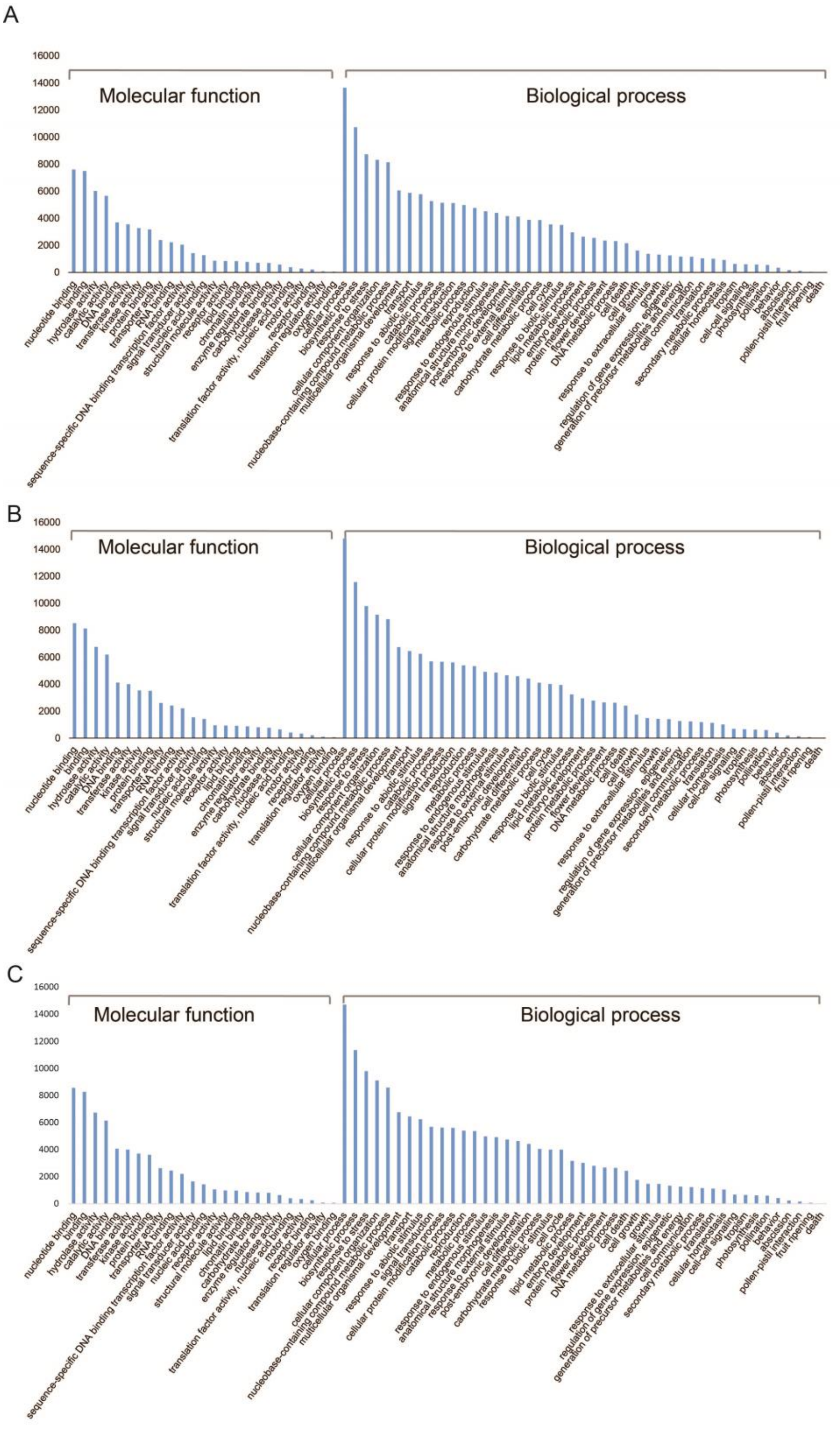

Addition file 1: GO term classification of assembled transcripts of three Chinese wild *Vitis*, *V. pseudoreticulata* accession “Baihe-13-1” (**A**), *V. pseudoreticulata* accession “Hunan-1” (**B**) and *V. quinquangularis* accession “Shang-24” (**C**).
